# Supplementary material for: A pharmacophore-guided deep learning approach for bioactive molecular generation
Source: Nat Commun. 2023 Oct 6;14:6234. doi: 10.1038/s41467-023-41454-9 (PMC10558534; doi:10.1038/s41467-023-41454-9)
Supplement: Supplementary file 1 — Supplementary Information [file 41467_2023_41454_MOESM1_ESM.pdf]

Supplementary Information: A Pharmacophore-Guided Deep Learning Approach for Bioactive Molecular Generation

Huimin Zhu[1,†], Renyi Zhou[1,†], Dongsheng Cao[2], Jing Tang[3, 4] and Min Li[1,*]

[1] School of Computer Science and Engineering, Central South University, Changsha 410083, China
[2] Xiangya School of Pharmaceutical Sciences, Central South University, Changsha 410008, China
[3] Research Program in Systems Oncology, Faculty of Medicine, University of Helsinki, Helsinki, 00290, Finland
[4] Department of Biochemistry and Developmental Biology, Faculty of Medicine, University of Helsinki, Helsinki, 00290, Finland

[†] These two authors contribute equally to the work.
* Corresponding author, limin@mail.csu.edu.cn

Supplementary Note 1: Pharmacophore description

We use *Basefeatures.fdef* (https://github.com/rdkit/rdkit/blob/master/Data/BaseFeatures.fdef) in RDKit[1], which contains a series of defined molecular substructures and their corresponding pharmacophore features, to obtain all the pharmacophore features of a molecule. The description and count for each pharmacophore feature are recorded in Supplementary Table 1. The typical pharmacophore features include Aromatic Ring, Cation (positive charge center), Hydrogen Bond Acceptors, Hydrogen Bond Donor, and hydrophobic group including Hydrophobe, Lumped Hydrophobe. As other types of pharmacophore features are rare, we label them as unknown.

Supplementary Table 1. Distribution of pharmacophore features and detailed description.

Type	Description	Counts on training set	Counts on pharmacophore	Occurrence rate on training set	Occurrence rate on pharmacophore
Aromatic Ring	Number of Π-electrons conforming to the 2n+2 rule and has no exocyclic double bonds	3148081	1153471	2.50	0.92
Cation	Atoms or functional groups ionized with formal positively charges	183953	505109	0.40	0.15
Hydrogen Bond Acceptor	Strongly electronegative atom that has at least one available 'lone pair' and formal charge of atom is not positive.	4958778	1768234	3.94	1.40
Hydrogen Bond Donor	Strongly electronegative atom that is covalently bonded to hydrogen atoms and its formal charge of atom	2525490	880398	2.01	0.700

	is not negative				
Hydrophobe	A group of continuous lipophilic contribution atoms (such as carbon atom) that are not connected to charged atoms or electronegative centers (non-ring)	5235429	1778375	4.16	1.41
Lumped-Hydrophobe	A group of contiguous lipophilic contribution atoms (such as carbon atom) not attached to an electrically charged atom or electronegativity center (ring)	2336036	833539	1.85	0.66
unknown	Rare pharmacophore features, such as Anion, Zinc Binder	283501	80726	0.22	0.064

* 'Counts on the training set' refers to statistics on all the pharmacophore features of the training set. 'Counts on pharmacophore' refers to statistics on all the pharmacophore features of the pharmacophore hypotheses in the training set. 'Occurrence rate on the training set' refers to the average frequency of a pharmacophore feature appearing in a molecule within the training set. 'Occurrence rate on a pharmacophore' refers to the average frequency of a pharmacophore feature appearing in a pharmacophore hypothesis.

Supplementary Note 2: Molecular pre-processing

During training, we perform the following four steps to extract a random pharmacophore from a molecule: 1) Use RDKit to identify all pharmacophore features within the molecule. 2) Select 3–7 pharmacophore features randomly; 3) Calculate the distances between the selected pharmacophore features by accumulating the relative bond lengths defined as Supplementary Table 2 along the shortest path on the molecular graph between two pharmacophore features. 4) The pharmacophore is coded as a complete graph, with the vertices representing the pharmacophore features and the edges representing the distances between two pharmacophore features.

Supplementary Table 2. Comparison of actual bond lengths with mapped bond lengths.

Covalent bond type	bond length (Å)	Relative bond length
Single bond	1.54	1.00
Double bond	1.34	0.87
Aromatic bond	1.38	0.91
Triple Bond	1.20	0.78

Supplementary Note 3: Analysis of the correlation and the differences between the shortest-path distance and the Euclidean distance

During the training process of PGMG, the distance used between pharmacophore points is the shortest-path distance rather than Euclidean distance. When the distances between pharmacophore features are represented using Euclidean distance, we linearly map them to the shortest path distances. To verify the feasibility of this

substitution, we conducted a comprehensive experiment using conformations from different sources, including docking conformations from the ePharmaLib database[2] (*ePharmaLib*), conformations retrieved from the PDBbind database[3] (*PDBbind*), and low-energy conformations calculated using RDKit (*RDKit*). We calculate the Euclidean distances between the pharmacophore features of these molecules, and aggregate relative bond lengths of bonds along the shortest path between two pharmacophore features and multiply the sum by 1Å to obtain the shortest-path distances with units.

For *ePharmaLib*, we obtained 15,148 pharmacophore hypotheses and corresponding molecules and conformations from the ePharmaLib database. The pharmacophores were modeled from solved protein-ligand complex structure initially extracted from the screening-PDB (sc-PDB)[4]. These complexes were then docked and scored with the Glide program[5], and the pharmacophoric features were selected according to the predicted binding energy terms. In the experiment, hypotheses with pharmacophoric features that did not match the types supported by RDKit were excluded. The shortest path distance and Euclidean distance were computed for each pharmacophore hypothesis. In total, 188,020 distance pairs and 13,055 pharmacophore hypotheses were utilized.

For *PDBbind*, we retrieved 19,442 molecules and molecular conformations from the PDBbind 2020 database. After filtering out files that failed to be read by RDKit, a total of 19,201 molecular conformations were obtained. We acquired a pharmacophore hypothesis for each molecular conformation by proportionally sampling the number and type of pharmacophore features, based on their occurrences in the ePharmaLib database. The shortest path distance and Euclidean distance were computed for each pharmacophore hypothesis. In sum, 260,864 distance pairs and 19,201 pharmacophore hypotheses were used.

For *RDKit*, we randomly selected 10,000 molecules from the ChEMBL database[6] and generated conformations using the ETKDG (Etoile's Triangle-Kekulé Distance Geometry) algorithm[7]. For each conformation, the pharmacophore hypothesis, the shortest path distance and Euclidean distance were acquired in a similar manner as *PDBbind*. Altogether, there were 133,360 distance pairs and 10,000 pharmacophore hypotheses.

As illustrated in Supplementary Figure 1, the shortest-path distances between pharmacophore features exhibit a strong correlation with the Euclidean distances across various sources of conformations with Pearson correlation coefficients of 0.924, 0.916, and 0.955, respectively.

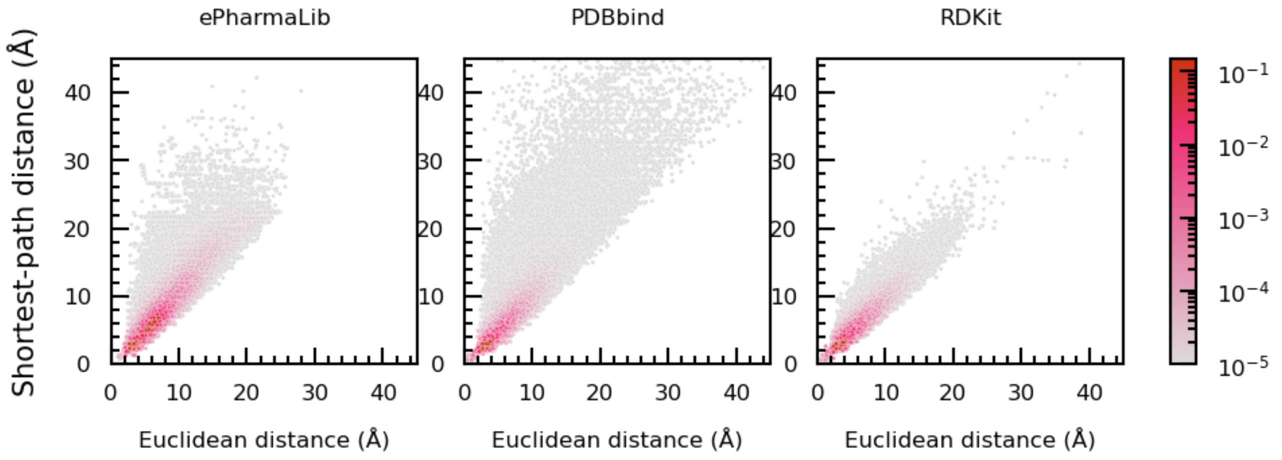

Supplementary Figure 1 | Euclidean and shortest-path distances between pharmacophore features. Molecular conformations are obtained from different sources: docking conformations from the ePharmaLib database (*ePharmaLib*), experimental conformations retrieved from the PDBbind database (*PDBbind*), and low-energy conformations calculated using RDKit (*RDKit*). The color bar represents the probability density calculated using a Gaussian kernel density estimation function.

Supplementary Figure 2 illustrates the distribution of the mean absolute differences between shortest-path distances and Euclidean distances within each pharmacophore hypothesis, and Supplementary Figure 3 illustrates the matching degree between shortest-path distances and Euclidean distances within each pharmacophore hypothesis. As illustrated in Supplementary Figure 2, the proportions of pharmacophore hypotheses with less than 1.5 Å absolute error between shortest-path and Euclidean distances are 79.0%, 83.2%, and 96.4%, respectively. Supplementary Figure 3 represents the Distribution of the matching degrees between the shortest-path distances and Euclidean distances within each pharmacophore hypothesis. The color of each bin represents the number of pharmacophore elements. The result shows that the level of inconsistence increases when the number of pharmacophore points increases, but the match degree is above 0.8 in most cases even under different sources of molecular conformations.

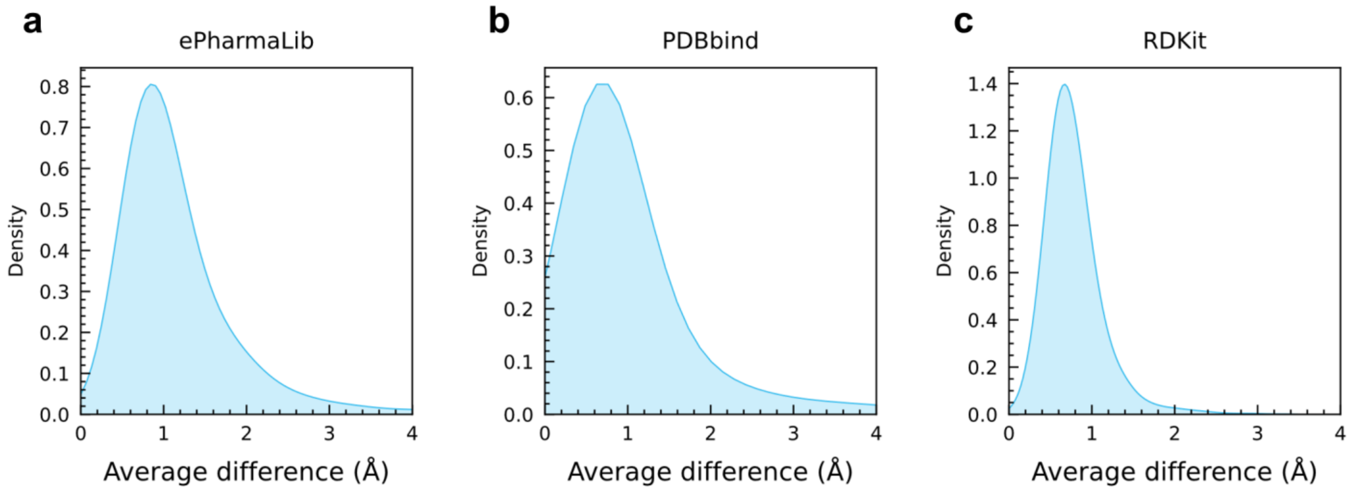

Supplementary Figure 2 | The mean absolute differences between Euclidean distances and shortest-path distances for various sources of conformations.

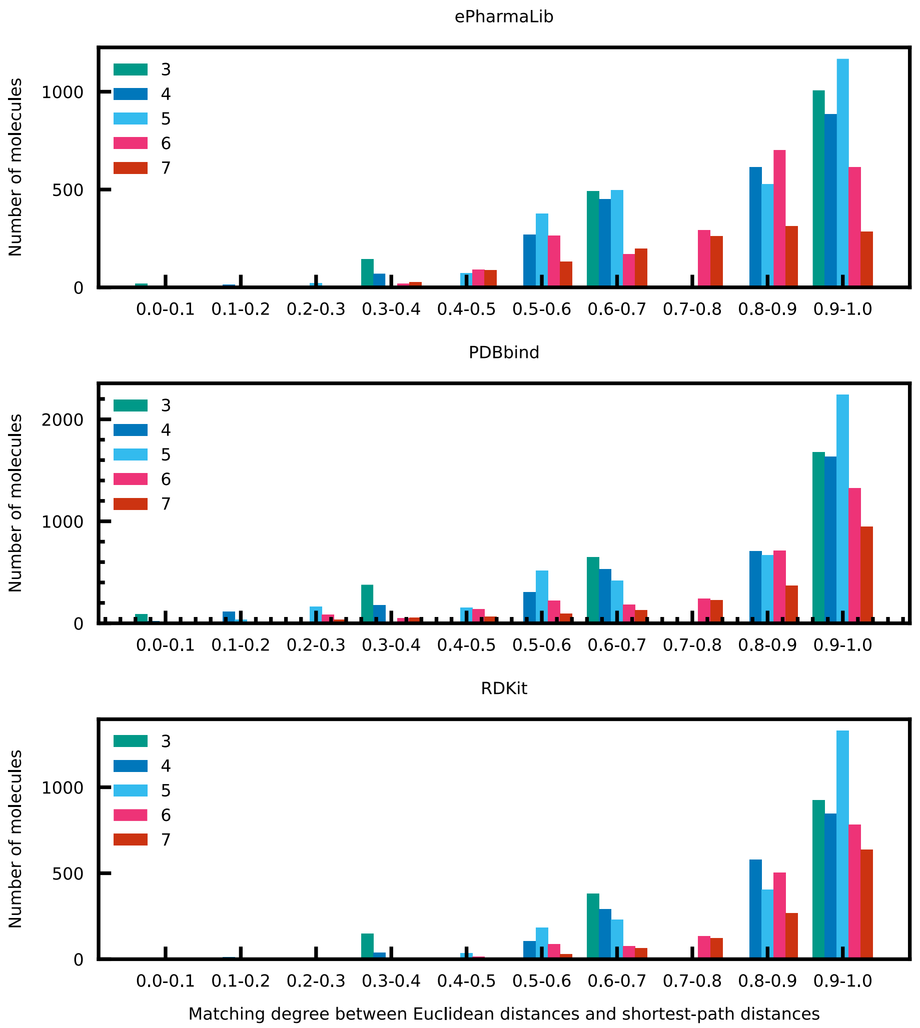

Supplementary Figure 3 | Distribution of the matching degrees between the shortest-path distances and Euclidean distances within each pharmacophore hypothesis. Bars of the same colour indicate a specific number of pharmacophore elements within a hypothesis. The matching degree is calculated as the percentage of distance pairs whose difference is less than 1.5Å.

The above experiments demonstrate the correlation between shortest-path distances and Euclidean distances. This correlation is helpful for constructing a practical shortest-path-distance-based pharmacophore constraint when the receptor structure is provided without any ligands. However, it should also be noted that the inconsistence between Euclidean distances and shortest-path distances does not imply that the generated molecules cannot match the given 3D pharmacophore hypothesis nor whether the generated molecules have activity or not.

Many generated molecules are likely to have a conformation that has a high overlap with the 3D pharmacophore hypothesis. To have a general idea of why, we can consider a ligand-receptor complex. A shortest-path-distance-based pharmacophore constraint and a Euclidian-distance-based pharmacophore constraint can be built upon the same pharmacophore hypothesis. The reference ligand sure satisfies both constraints and is therefore may be retrieved by generative methods that use either constraint. This holds true for many other conceivable molecules, even if the shortest-path distances are not consistent with the Euclidean distances. For example, in Supplementary Figure 4, the generated molecules (green) are able to fold

themselves to match the 3D pharmacophore hypotheses as the reference molecules (white).

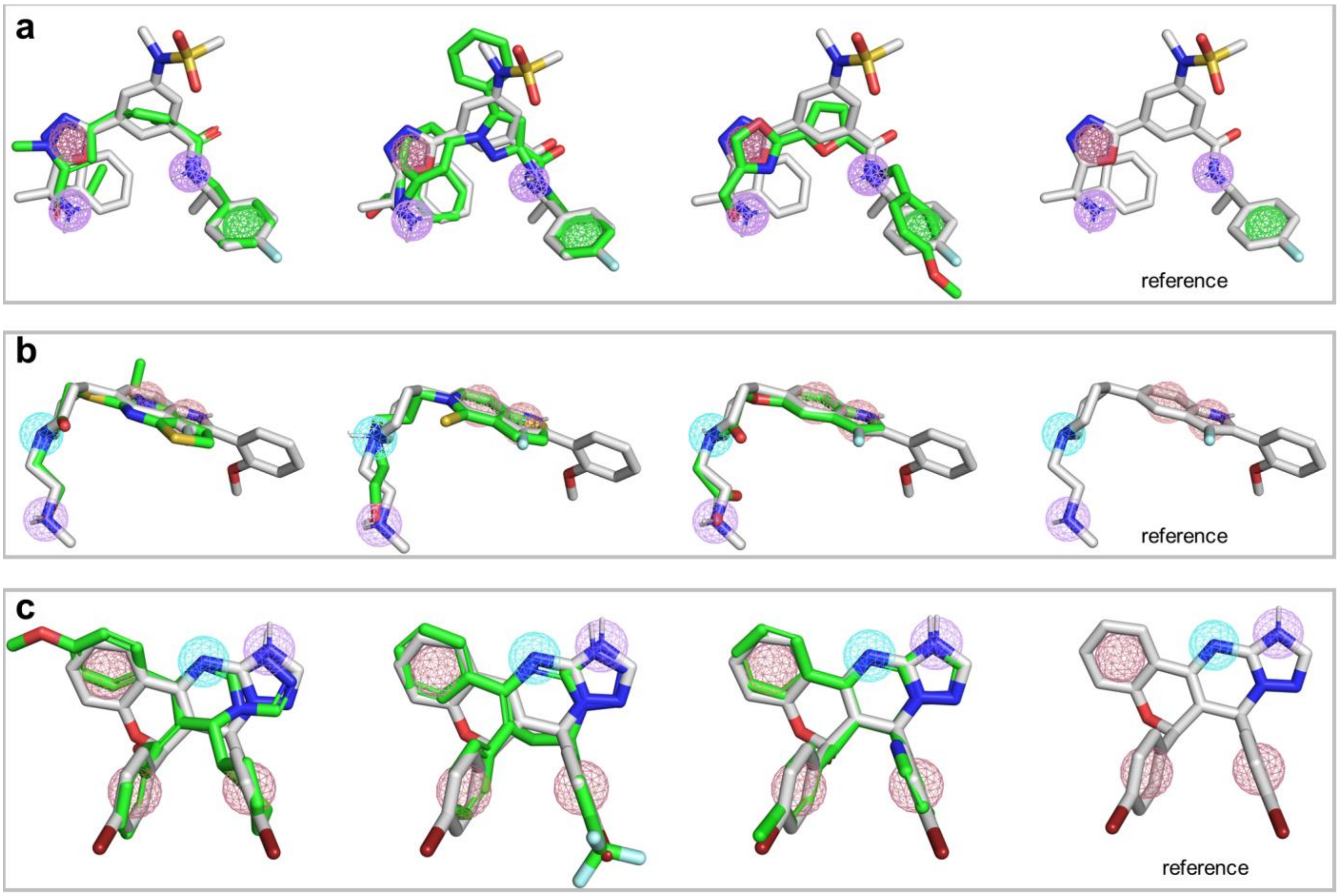

Supplementary Figure 4 | Alignment conformations of reference molecules (white) and generated molecules (green) with the pharmacophore hypotheses. (a), (b) and (c) represent different pharmacophore hypotheses, where the conformations of the reference molecules are obtained from the PDB complexes (a) 2IRZ, (b) 2Y1W and (c) 3JZK. The coloured spheres represent different pharmacophore elements: aromatic ring (red), hydrophobic group (green), hydrogen bond donor (purple) and hydrogen bond acceptor (blue).

There are pros and cons of using shortest-path distances instead of Euclidian distances. One disadvantage is that molecules matching the 3D pharmacophore hypothesis may have multiple mode of the relative positions of pharmacophore elements within the molecules. For example, for a 3D pharmacophore hypothesis with three different pharmacophore elements placed at the endpoints of an equilateral triangle, these pharmacophore points can be connected in multiple ways within a molecule, such as ">" or "V", which corresponds to different shortest-path-distance based pharmacophore constraints. But the impact of this disadvantage is also influenced by the target structure. For instance, there could be some invalid ways to connect and ignoring them is only beneficial. This disadvantage lies mainly in the structure-based drug design scenario. In the ligand-based drug design scenario, we can generate new shortest-path-distance based pharmacophore constraints if there are very different backbones in the collected ligands. Besides, when using ligands to build pharmacophore hypothesis without the knowledge of the receptor structure, it is difficult to determine what could be the active conformations. Using shortest-path distance to encode the relative positions of pharmacophore elements may be a simple solution to avoid conformational search which makes PGMG less computationally demanding.

Supplementary Note 4: Calculation of match score

A molecule may have many pharmacophore features and a subset of them can be used to construct a pharmacophore. We can convert all the pharmacophore features of a molecule into a fully connected graph

$$G_f = \{V_f, E_f\}$$

with a node set $V_f = \{v_{f_1}, v_{f_2}, \dots, v_{f_n}\}$ and an edge set $E_m = \{e_{f_{1,2}}, e_{f_{1,3}}, \dots, e_{f_{1,n}}, e_{f_{2,3}}, e_{f_{2,4}}, \dots, e_{f_{n-1,n}}\}$. And a pharmacophore be turned into a graph G_p in the same way. Then the problem of calculating the matching degree between a given molecule and a given pharmacophore can be seen as finding the best match of a small graph in a large graph. Since molecules that we deal with usually contain a small number of heavy atoms, we simply use brute force to calculate the match score. The calculation steps are as follows:

Input: G: The pharmacophore graph to be matched; SMILES: SMILES that need to be verified.

Output: The matching score for Pharmacophore and SMILES

MATCH SCORE (G, SMILES)

```
V_r = G.nodes()
E_r=G.edges()
Extract chemical features from SMILES using RDKit
Transform SMILES into a graph G_Q(V_q, E_q) with chemical features as nodes
type_list = [ ]
score_list = [ ]
for i = 1 to length(V_r):
    type = [ ]
    for j=1 to length(V_r):
        if ref_type[i] == V_r[j]:
            type.append(V_r[j])
    type_list.append(type)
for k = 1 to (length(type_list[0])* (length(type_list[1])*…* (length(type_list[-1]):
    dist_true=0
    Extract one node and corresponding edge from different type_list at a time to get a candidate
      subgraph G_k(V_k, E_k)
    for l = 1 to length(V_r):
        for m = 1 to length( V_r):
            if m≠l and |E_k(l, m)-E_r(l, m)|<1.2:
                dist_true = dist_true+1
```

```
        score=dist_true/length(E_k)
        score_list.append(score)
    return max(score_list)
```

To make the calculation process clearer, we give some examples about the calculation of the match score. The pharmacophore in Supplementary Figure 5a contains four pharmacophore features, where green indicates the hydrophobic group, red indicates the aromatic ring, and blue indicates the hydrogen bond acceptors. Supplementary Figure 5b, Supplementary Figure 5c, and Supplementary Figure 5d give three molecules generated by PGMG with match scores of 1.0, 0.5, and 0.5, respectively. The molecule in Supplementary Figure 5b is an example of a perfect match, while the molecules in Supplementary Figure 5c and Supplementary Figure 5d have their problems. In Supplementary Figure 5c, the hydrogen bond acceptor formed by the carbonyl group is too far from the other pharmacophore features. In Supplementary Figure 5d, the molecule lacks a hydrogen bond acceptor and therefore can't match the lower right hydrogen bond acceptor in Supplementary Figure 5a.

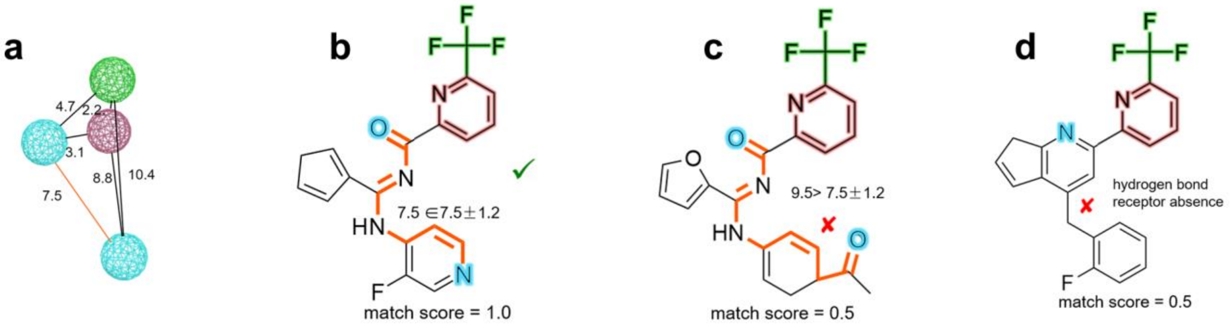

Supplementary Figure 5 | Illustration of the matching score calculation process. (a) A pharmacophore. (b) A molecule satisfying given pharmacophores. (c), (d) molecules unsatisfying given pharmacophores.

Supplementary Note 5: Ablation Study

In the ablation study, we remove features of PGMG and see how that affects performance. All models are trained using the ZINC[8] dataset with the same parameters. To evaluate validity, uniqueness, and novelty, a pharmacophore hypothesis is extracted from each of the 208, 987 molecules in the test dataset, and for each hypothesis, 10 molecules are generated. The match score is evaluated by generating 512×512 molecules for 512 pharmacophore hypotheses randomly sampled from molecules in the test dataset.

As shown in Supplementary Table 3, when using canonical SMILES to train PGMG ("canonical_SMILES"), the uniqueness increases from 0.976 to 0.991, but the match score decreases from 0.935 to 0.914. A similar result can be found when we change the Gaussian distribution of the latent variable z to a Dirac delta distribution, denoted as PGMG ("remove_z"). "remove_z" exhibits a huge decrease on the uniqueness (from 0.976 to 0.806) and a certain degree of increase on the match score (from 0.935 to 0.969). In the generation process, the model will produce a discrete probability score distribution of the next token given the latent variable z and the formerly generated tokens. By default, for stability, the next token chosen by PGMG is

the token with the highest score. But the next token can also be chosen by sampling the multinomial distribution defined by the scores (*random sampling*). With *random sampling*, we can increase the uniqueness of generated molecules, but it cannot make up for the drop of both the validity and the match score. From these results, there appears to be an intuitive trade-off between the uniqueness and match score.

Supplementary Table 3. Results of the ablation study.

	**Validity**	**Uniqueness**	**Novelty**	**Ratio of available molecules**	**Match score ±std**
PGMG	0.972	0.976	0.998	94.8%	0.935±0.152
PGMG(canonical_SMILES)	0.976	0.991	0.997	**96.4%**	0.914±0.171
PGMG (remove_dis)	**0.990**	0.823	0.996	81.2%	0.592±0.208
PGMG (remove_z)	0.991	0.806	0.996	79.6%	**0.969±0.108**
PGMG(remove_z, random sampling)	0.917	**0.999**	**0.999**	91.5%	0.886±0.196

* Best performance among all methods for each metric is shown in bold. "std": the standard deviation.

We also test PGMG's performance when replacing the distance between chemical features with a constant number PGMG ("remove_dis"). As expected, we see a large decrease in both uniqueness (from 0.98 to 0.82) and match score (from 0.935 to 0.592), which suggests that the spatial information of pharmacophores is an important feature for PGMG prediction.

Supplementary Note 6: Docking result of Vina

Supplementary Table 4 shows the average docking score calculated using AutoDock Vina[9] of the top 1000 generated molecules and the docking score of the activity molecules with the specific targets obtained from the ChEMBL database. We calculate the RMSD of the top conformations of the reference ligand and the bioactive conformation acquired from PDB[10]. And the RMSD shows that the top conformations of the reference ligand are relatively close to the active conformations acquired from PDB (Supplementary Figure 6), which indicates the reliability of our docking results. The IC50 values of the reference ligands for specific targets are also listed in the table to facilitate a comparison of the molecule's inhibition of the target. It is also worth mentioning that the IC50 values of the four reference ligands corresponding to the proteins are all at the nanomolar level, and the same level of affinity of the generated molecules implies that the molecules generated by PGMG are also likely to have good biological activity.

Supplementary Table 4. molecular docking results of different receptors generated by PGMG.

target	PDB ID	average docking score of	average docking score of	docking score of	RMSD (Å)	IC50 of reference

		top 1000 molecules±std (kcal/mol)	bioactivity molecules±std (kcal/mol)	reference ligand (kcal/mol)		ligand (nM)
FGFR1	2FGI	-9.17±0.36	-8.33±0.80	-7.3	1.34	21
ACHE	4EY7	-12.63±0.47	-10.22±1.54	-12.2	0.42	43
MDM2-P53	3JZK	-9.59±0.39	-7.92±1.01	-9.5	0.20	1230
PARP1	6I8M	-11.08±0.55	-10.04±1.53	-10.6	0.66	50
NS5B	3PHE	-10.12±0.31	-7.84±0.97	-8.0	1.14	16
HSP90	3HHU	-9.07±0.36	-7.66±1.00	-9.3	0.58	41
BACE1	2IRZ	-10.50±0.37	-8.35±1.13	-12.4	0.24	12
PRKCQ	1XJD	-10.37±0.49	-8.91±1.16	-13.3	0.18	0.33
PIM1	3BGQ	-10.94±0.50	-9.62±0.98	-10.3	0.79	11
CDK2	4KD1	-9.48±0.40	-8.03±0.92	-9.6	0.68	-
BRD4	3MXF	-8.84±0.38	-7.22±0.77	-9.1	0.80	49
VEGFR2	1YWN	-9.60±0.41	-8.12±1.06	-10.8	0.59	3
CDK6	2EUF	-10.96±0.48	-9.67±0.91	-10.5	1.36	15
TGFB1	6B8Y	-10.81±0.45	-9.82±0.99	-11.5	0.45	0.56
AKT1	4GV1	-11.53±0.42	-9.53±1.07	-10.7	0.76	3

* “std”: the standard deviation.

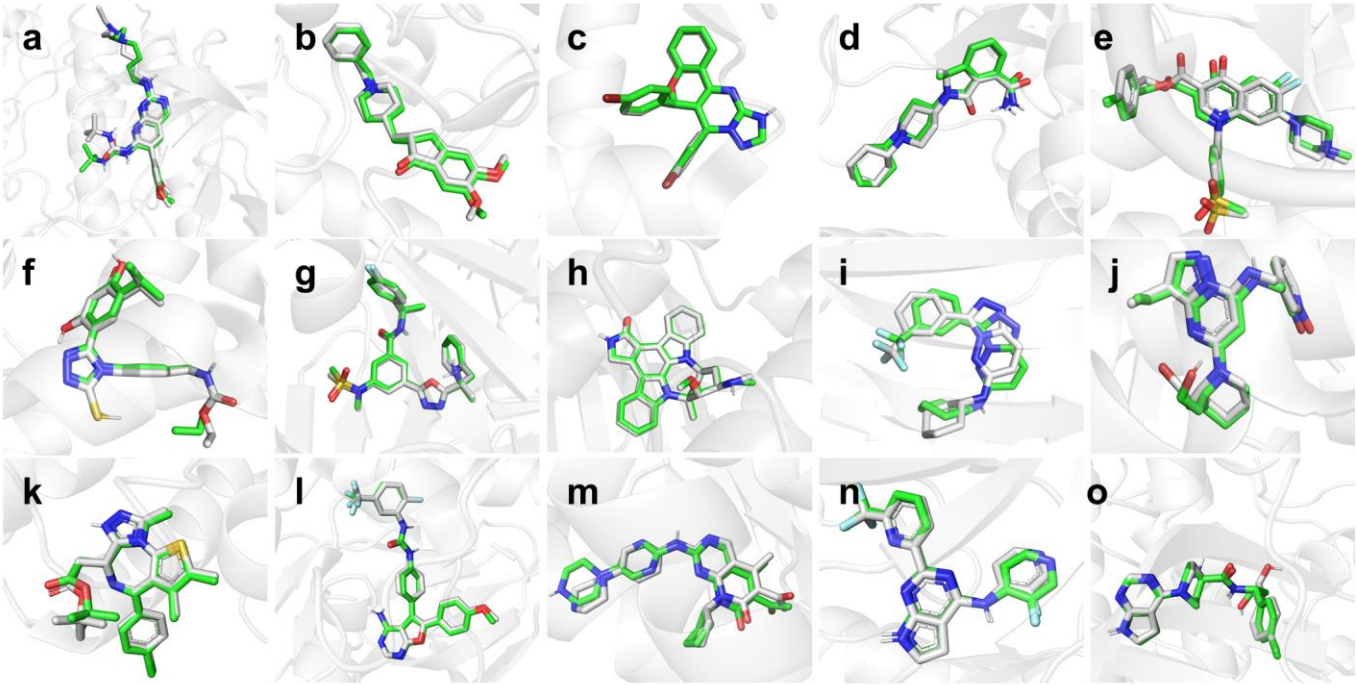

Supplementary Figure 6 | A display of the fifteen reference ligand conformations in the PDB, and the top affinity conformation using vina docking in the protein pocket. The conformation in gray is the reference ligand of PDB, the conformation in green is acquired by AutoDock Vina. (a) FGFR1 (2FGI), (b) ACHE (4EY7), (c) MDM2-P53 (3JZK), (d) PARP1 (6I8M), (e) NS5B (3PHE), (f) HSP90 (3HHU), (g) BACE1 (2IRZ), (h) PRKCQ (1XJD), (i) PIM1 (3BGQ), (j) CDK2 (4KD1), (k) BRD4 (3MXF), (l) VEGFR2 (1YWN), (m) CDK6 (2EUF), (n) TGFB1 (6B8Y), (o) AKT1 (4GV1).

Supplementary Note 7: Pharmacophore-guided docking

Besides docking with AutoDock Vina, we also conducted pharmacophore-guided docking experiments utilizing the Molecular Operating Environment (MOE)[11], in which the docking pose of a molecule is constrained by the given pharmacophore hypotheses. Specifically, we performed these experiments on the previously generated molecules with a match score greater than 0.85. We presented the top 1000 molecules based on their docking scores. As a comparison, we collected active molecules (pChEMBL > 4) from the ChEMBL database and performed pharmacophore-guided docking experiments. In addition, the docking results of known active molecules without pharmacophore guidance were shown as a reference. Molecules that do not match the given 3D pharmacophore hypothesis or collides with the pocket are filtered out. The results are illustrated in Supplementary Figure 7. The docking results demonstrated that the generated molecules by PGMG exhibited comparable performance. This indicates that a considerable portion of the generated molecules maintained the pharmacophore interaction and possess strong affinity.

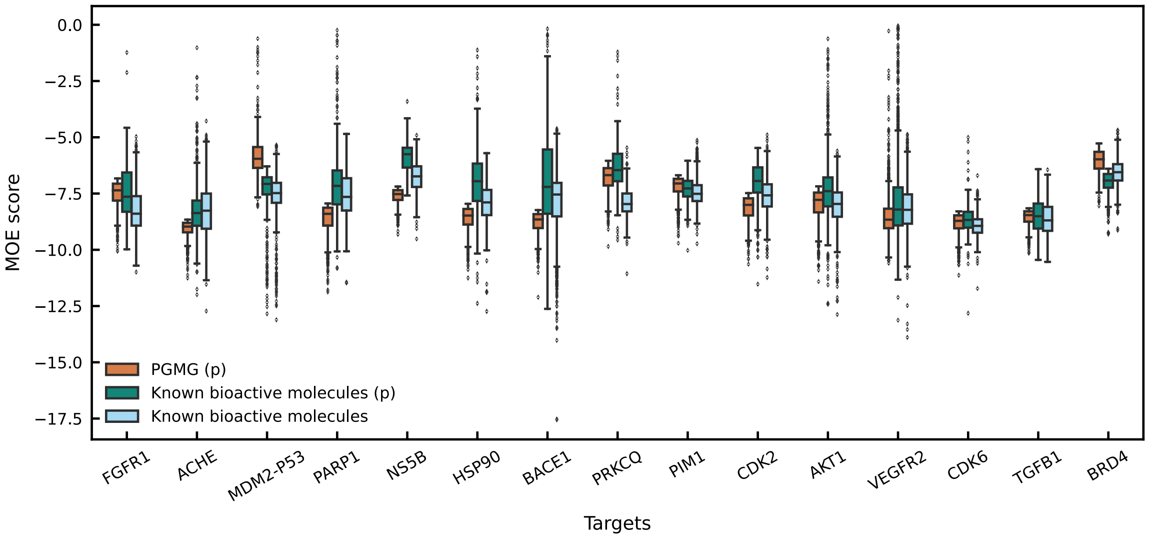

Supplementary Figure 7 | Box plots of the docking scores for 15 targets. PGMG (p) represents pharmacophore-guided docking scores of the top 1,000 molecules generated by PGMG. Know Bioactive Molecules (p) represents pharmacophore- guided docking scores of the top 1,000 active molecules (pChEMBL > 4). The pChEMBL value is the negative logarithm of the molar IC50, EC50, Ki, Kd, or Potency, and it allows these roughly comparable measures to be compared. Known Bioactive Molecules represent the docking results of all active molecules without pharmacophore guidance. The median is represented by the centerline of the boxplot, while the first and third quartiles are indicated by the bounds of the box. The whiskers represent the 1.5 interquartile range (IQR).

We used the general mode of MOE to perform pharmacophore-guided docking. Molecules that do not match the given 3D pharmacophore hypothesis or collides with the pocket are filtered out. For active molecules

without pharmacophore-guidance, the "Triangle Matcher" method is used for Placement, while other settings remain the same as for the pharmacophore-guided docking.

Below is the detailed description of the experiment setting:

1. Protein preparation: The protein preprocessing was facilitated through the application of the QuickPrep in MOE. Water molecules were removed prior to docking for all targets except for BRD4.
2. Pharmacophore preparation: The radius of the pharmacophore was designated at 1.5 Å.
3. Ligand preparation: The 'wash' module was utilized to process the molecules and to facilitate the generation of 3D conformations.
4. Docking parameter settings: The binding site of the ligand in the protein-ligand complex was used as the known sites for docking. The placement was executed via the 'pharmacophore' method, utilizing the London dG scoring function and resulting in the generation of 30 conformations per search. During the docking process, the GBVI/WSA dG scoring function was employed, and five top poses to be retained

Supplementary Note 8: Properties distribution of PGMG-generated molecules and active molecules

To assess the drug-like properties of generated molecules comprehensively, we use ADMETlab2.0[12] to calculate the absorption, distribution, metabolism excretion and toxicity (ADMET) properties of 15,787 active molecules from the ChEMBL database and 131, 533 molecules generated according to fifteen protein structures. We leave out indicators in the ADMETlab that are not given explicit thresholds and obtain 36 ADME indicators and 29 toxicity indicators.

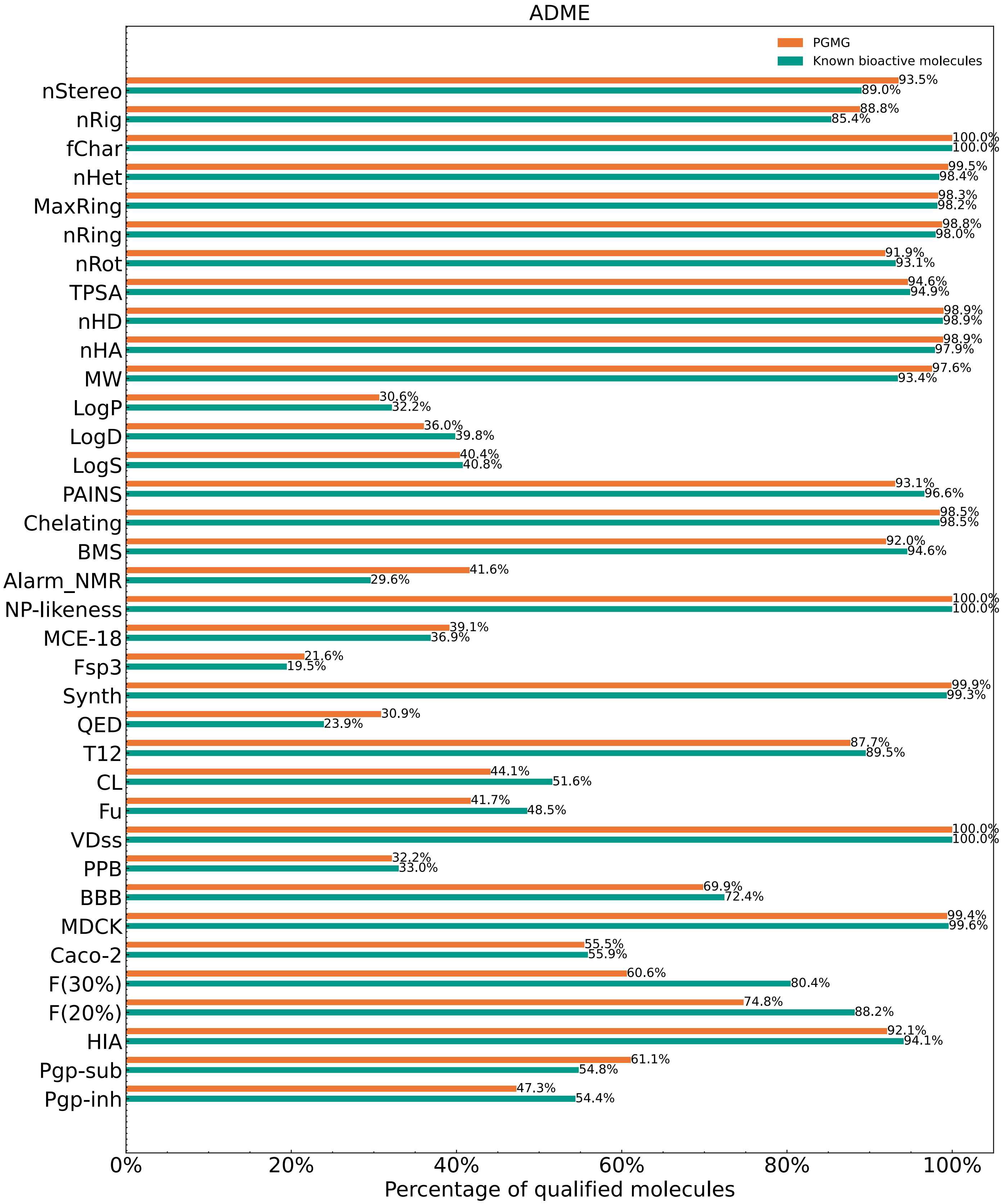

Supplementary Figure 8 | Percentage of qualified molecules of generated molecules and the known active molecules in terms of absorption, distribution, metabolism, excretion (ADME) properties. The properties and their respective threshold values can be found in ADMETlab2.0. Among them, "NP-likeness" stands for "Nature Products-likeness."

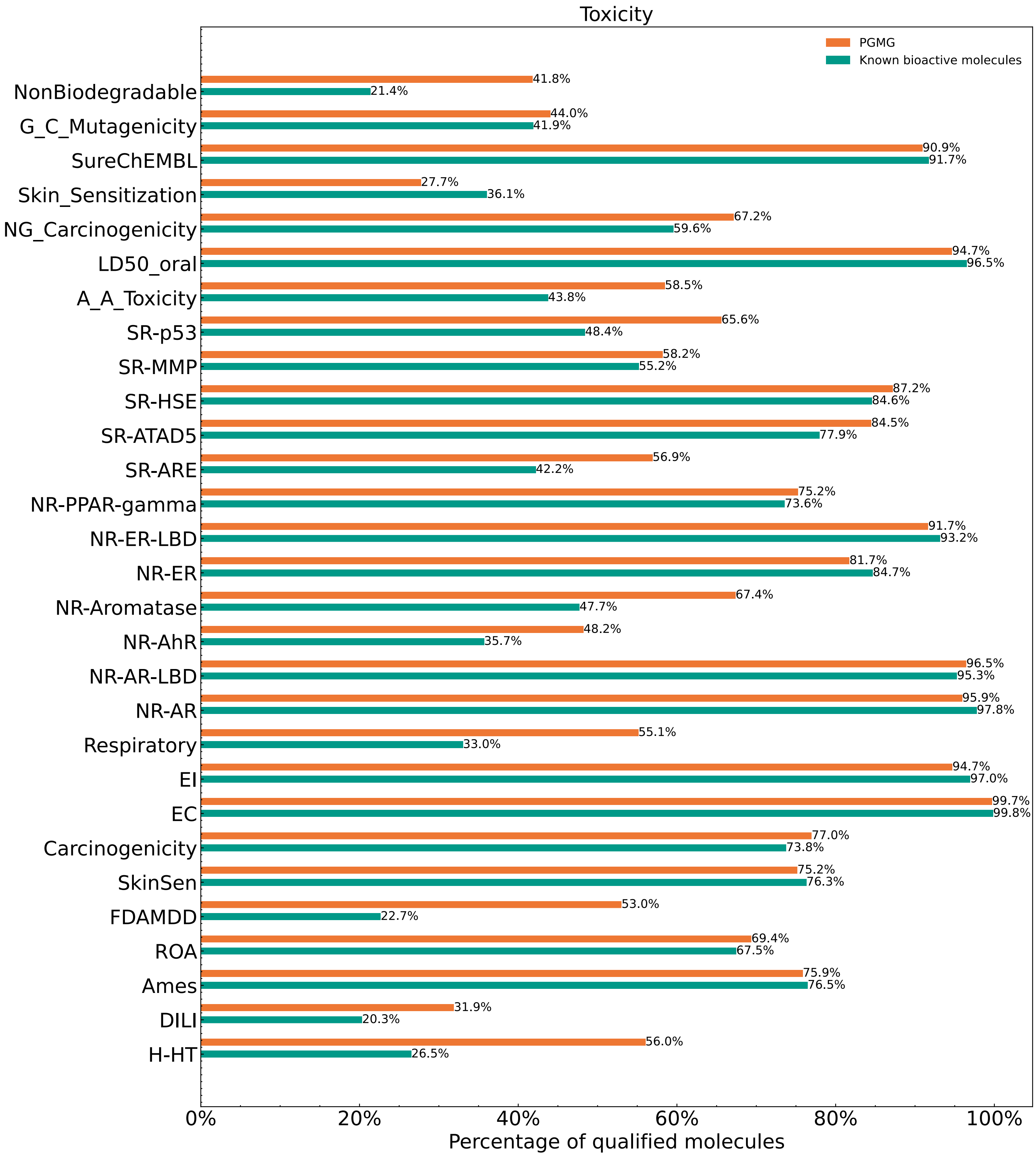

Supplementary Figure 9 | Percentage of qualified molecules of PGMG-generated molecules and the known active molecules in terms of toxicological indicators. The properties and their respective threshold values can be found in ADMETlab2.0. Among them, "NG_Carcinogenicity" stands for "NonGenotoxic_Carcinogenicity"; "G_C_Mutagenicity" stands for "Genotoxic_Carcinogenicity_Mutagenicity".

Supplementary Figure 8 presents a comparison of the ADME predicted properties between the generated molecules and known active molecules, showing the percentage of molecules that meet the ADME criteria. Overall, the molecules generated by PGMG behave comparably to the active molecules.

Supplementary Figure 9 shows the percentage of qualified molecules that meet the toxicological criteria. The above results demonstrate that PGMG has the ability to generate drug-like molecules.

Supplementary Note 9: Analysis of the binding sites of the generated molecules in EGFR.

In the 'A showcase of PGMG application in scaffold hopping' section, we demonstrate that PGMG is capable of generating compounds novel scaffolds compared to EGFR inhibitors. Furthermore, we have added new analysis of the binding regions of these generated molecules. These generated molecules have the same aromaticity, hydrophobic region (Leu694, Lys721) and hydrophilic region (Met769) as Lavendustin A[13]. This implies that the generated molecules can have the same binding mode restricted by the given pharmacophore as Lavendustin A. The high similarity to the EGFR bioactive molecules and consistent binding site with Lavendustin A of the generated molecules indicates that PGMG can discover these inhibitors that have novel scaffolds with only the knowledge of Lavendustin A.

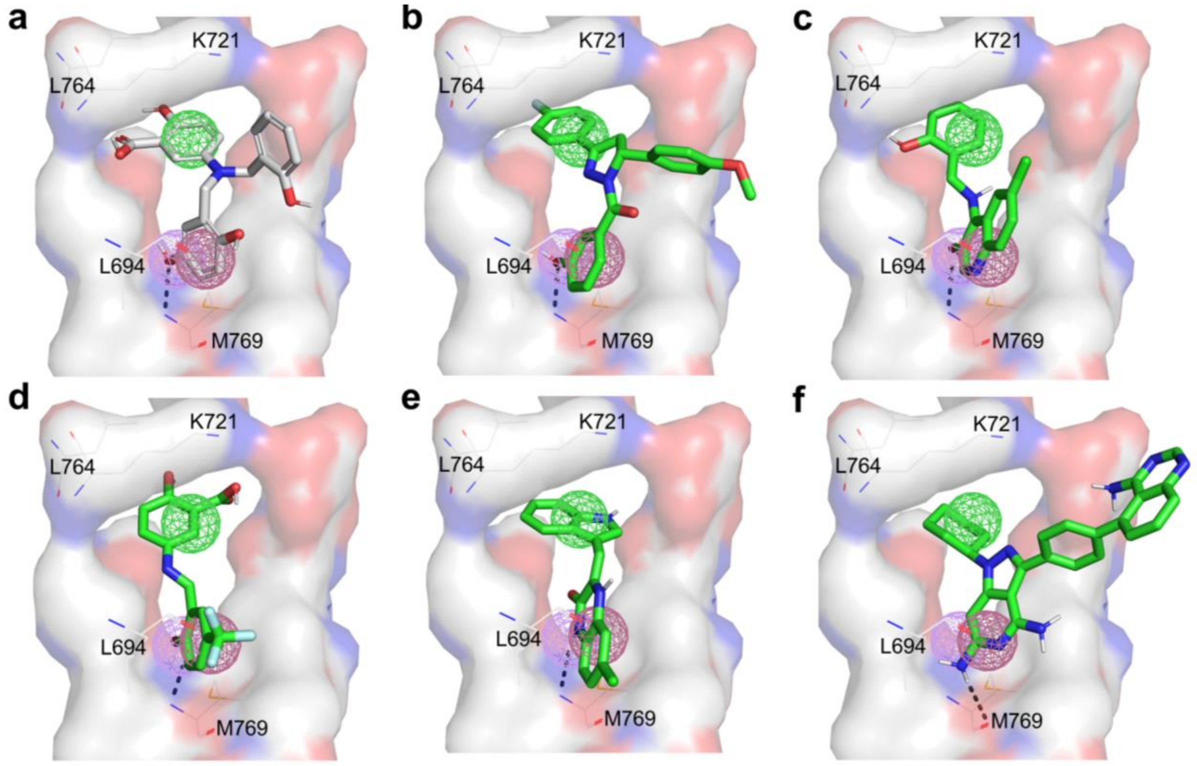

Supplementary Figure 10 | The binding surface of Lavendustin A and the generated molecules in the pocket of EGFR (1M17). (a) Lavendustin A, (b-f) generated molecules by PGMG. The different coloured spheres represent different pharmacophore features: aromatic ring (red), hydrogen bond donor (purple) and hydrophobic group (green). Lys721 (K721), Leu764 (L764), Leu694(L694) and Met769(M769) represent amino acid residues from the crystal structure of EGFR (1M17).

Supplementary Note 10: Masked mapping scores and labels

Since the SMILES format contains tokens other than atom symbols, we mask them when calculating the mapping loss. The mapping loss is then calculated as the cross-entropy of the masked scores and labels. An illustration of the masked mapping score and label is given in Supplementary Figure 11.

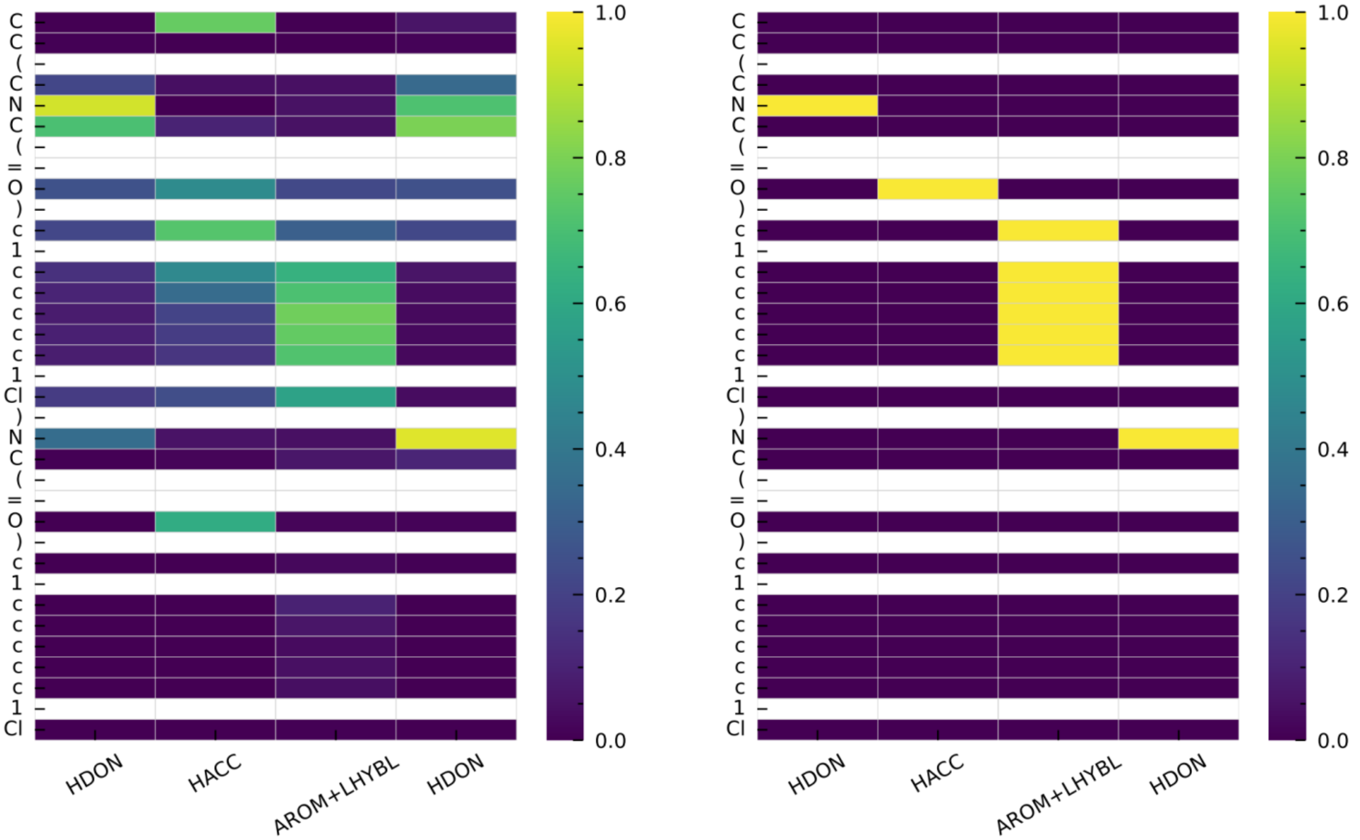

Supplementary Figure 11 | An illustration of masked mapping scores and labels. Each row represents a SMILES token of a molecule and each column represents a pharmacophore feature. The SMILES tokens and pharmacophore types are shown alone the x-axis and y-axis. Tokens that are not heavy atoms are masked and denoted in white. The color indicates the mapping scores between heavy atoms and pharmacophore features. (a) the predicted mapping scores. (b) the ground truth mapping scores.

Supplementary References

1. Landrum, G. http://www.rdkit.org.
2. Moumbock, A.F. et al. ePharmaLib: A Versatile Library of e-Pharmacophores to Address Small-Molecule (Poly-) Pharmacology. *Journal of Chemical Information and Modeling* **61**, 3659-3666 (2021).
3. Liu, H., Su, M., Lin, H.-X., Wang, R. & Li, Y. Public data set of protein–ligand dissociation kinetic constants for quantitative structure–kinetics relationship studies. *ACS omega* **7**, 18985-18996 (2022).
4. Desaphy, J., Bret, G., Rognan, D. & Kellenberger, E. sc-PDB: a 3D-database of ligandable binding sites—10 years on. *Nucleic acids research* **43**, D399-D404 (2015).
5. Friesner, R.A. et al. Glide: a new approach for rapid, accurate docking and scoring. 1. Method and assessment of docking accuracy. *Journal of medicinal chemistry* **47**, 1739-1749 (2004).
6. Mendez, D. et al. ChEMBL: towards direct deposition of bioassay data. *Nucleic acids research* **47**, D930-D940 (2019).
7. Riniker, S. & Landrum, G.A. Better informed distance geometry: using what we know to improve conformation generation. *Journal of chemical information and modeling* **55**, 2562-2574 (2015).

8. Sterling, T. & Irwin, J.J. ZINC 15–ligand discovery for everyone. *Journal of chemical information and modeling* **55**, 2324-2337 (2015).
9. Trott, O. & Olson, A.J. AutoDock Vina: improving the speed and accuracy of docking with a new scoring function, efficient optimization, and multithreading. *Journal of computational chemistry* **31**, 455-461 (2010).
10. Burley, S.K. et al. Protein Data Bank (PDB): the single global macromolecular structure archive. *Protein Crystallography* **1607**, 627-641 (2017).
11. Corbeil, C.R., Williams, C.I. & Labute, P. Variability in docking success rates due to dataset preparation. *Journal of computer-aided molecular design* **26**, 775-786 (2012).
12. Xiong, G. et al. ADMETlab 2.0: an integrated online platform for accurate and comprehensive predictions of ADMET properties. *Nucleic Acids Research* **49**, W5-W14 (2021).
13. Żołek, T., Trzeciak, A. & Maciejewska, D. Theoretical evaluation of EGFR kinase inhibition and toxicity of di-indol-3-yl disulphides with anti-cancer potency. *Journal of Biomolecular Structure and Dynamics* **40**, 622-634 (2022).
