# Supplementary material for: A pharmacophore-guided deep learning approach for bioactive molecular generation
Source: Nat Commun. 2023 Oct 6;14:6234. doi: 10.1038/s41467-023-41454-9 (PMC10558534; doi:10.1038/s41467-023-41454-9)
Supplement: Supplementary file 3 — Description of Additional Supplementary Files [file 41467_2023_41454_MOESM3_ESM.pdf]

Description of additional Supplementary files

File name: Supplementary Data1
Description:

Figure 3

The "Figure 3" folder contains two files: "random_SMILES.csv," which includes randomly selected molecules' SMILES along with their corresponding match scores displayed in Figure 3, and "PGMG_SMILES.csv", which contains SMILES of molecules generated by PGMG along with their corresponding match scores.

1.1 Table 2

The folder “Table 2.” contains the synthetic accessibility (SA) scores, docking scores, and pharmacophore scores (if applicable) of molecules generated by various methods as presented in Table 2.

1.2 SMILES of 15 targets generated by PGMG

The Excel file "SMILES of 15 targets generated by PGMG.xlsx" contains the SMILES of molecules generated by PGMG for the 15 targets showcased in Figure 4a.

1.3 SMILES of 15 targets obtained from ChEMBL

The Excel file "SMILES of 15 targets obtained from ChEMBL.xlsx" contains the SMILES of active molecules collected from the ChEMBL database for the 15 targets showcased in Figure 4a.

File name: Supplementary Data2
Description:

1.4 Supplementary Table 3

The file "Match score of Ablation Study.csv" records the match scores of different methods in the ablation study.

1.5 Supplementary Table 4

The file "Supplementary Table 4.csv" contains the top 1000 docking scores for both the molecules generated by PGMG and the active molecules obtained from the ChEMBL database corresponding to the 15 targets used in Figure 4.

File name: Supplementary Data3
Description:

1.6 ChEMBL_ADMET

The file "ChEMBL_ADMET.csv" presents the results of the ADMET predictions for active molecules collected from the ChEMBL database corresponding to these 15 targets. This file contains the raw data for the predicted ADMET properties of active molecules as presented in Supplementary Figure 8 and Supplementary Figure 9.

1.7 PGMG_ADMET

The file "PGMG_ADMET.csv" displays the results of ADMET predictions for molecules generated by PGMG targeting these 15 targets. This file contains the raw data for the predicted ADMET properties of PGMG-generated molecules as presented in Figure 4b, Supplementary Figure 8, and Supplementary Figure 9.
